# Targeted analysis of sphingolipids and cytokines in plasma of dairy cows after calving reveals distinct impacts of systemic inflammation, ketosis, and mastitis

**DOI:** 10.1186/s40104-025-01325-3

**Published:** 2026-01-12

**Authors:** Elodie Lassallette, Alix Pierron Baysse, Blandine Gausseres, Gilles Foucras, Philippe Guerre

**Affiliations:** 1 IHAP, Université de Toulouse, INRAE, ENVT, Toulouse, 31076 France; 2 Olmix S.A., ZA du Haut du Bois, Bréhan, 56580 France

**Keywords:** Cytokines, Dairy cow, Disease, Postpartum, Sphingolipids

## Abstract

**Background:**

Sphingolipids (SL) are key regulators of inflammatory processes, yet their roles in dairy cows remain poorly understood. This study investigated the effects of inflammation (plasma haptoglobin concentration), ketosis, and mastitis on plasma SL profiles in Holstein cows sampled seven days postpartum. From a cohort of 427 cows across 25 farms, 80 animals were classified into four groups: inflammation (*n* = 20), ketosis (*n* = 19), mastitis (*n* = 21), and healthy controls (*n* = 20). Plasma SL were quantified by targeted HPLC–MS/MS, while cytokines were quantified with a 15-plex bead-based assay. Both univariate and multivariate analyses were applied to assess pathological effects, along with SL ratios and correlations between SL and cytokines.

**Results:**

Systemic inflammation detected through the haptoglobin measure induced the most pronounced alterations in SL metabolism, characterized by elevated dihydrosphingomyelins (DHSM) and lactosylceramides (LacCer), higher C22–24:C16 ratios, and lower unsaturated:saturated ratios in ceramides (Cer) and sphingomyelins (SM). Although total Cer, SM, and the Cer:SM ratio remained unchanged, specific reductions were observed in both Cer and SM in C14, Cer C18:1, SM C16:1, and SM C23:1, whereas SM C25:0 and C26:0 increased. Sphingosine-1-phosphate (So1P) was positively correlated with IL-10 as well as IL-1α and TNFα, while C18–20 Cer correlated positively with multiple pro-inflammatory cytokines and chemokines such as CXCL8 and CCL2. Ketosis induced subtler changes, primarily an increase in plasma DHSM and DHSM:SM ratio (driven by C16:0), an increase in C22–24:C16 DHCer ratio, and a decrease in both LacSo:LacCer and unsaturated:saturated ratios in C23-SM. In this group, So1P correlated positively with CXCL8 and CCL2. Moreover C18–20 Cer and DHCer were positively associated with CXCL8, CCL2, CCL3, and CCL4, which also showed correlations with most LacCer species. Analysis of chronic mastitis cases yielded a clear separation from controls in multivariate analysis but only minimal changes in SL concentrations and ratios, maybe due to the localized nature of the inflammatory response.

**Conclusions:**

In summary, heightened inflammatory response in early post-partum is associated with the strongest systemic effects on SL metabolism, followed by ketosis, while mastitis induced only modest alterations. These findings highlight condition-specific patterns of SL regulation postpartum and suggest potential immunometabolic biomarkers of disease.

**Supplementary Information:**

The online version contains supplementary material available at 10.1186/s40104-025-01325-3.

## Background

In dairy cows, nearly 75% of the diseases occur during the peripartum period, making this stage the highest-risk window for morbidity and mortality [1]. Calving itself induces an acute inflammatory response in the days following parturition, activating the immune system but simultaneously increasing the cow’s susceptibility to disease [2]. Metabolic disorders such as ketosis, which commonly arise in early lactation, further compromise immunity and predispose cows to infection [3]. Among infectious diseases, mastitis represents a major health and economic burden in dairy production [4], with its occurrence determined by both pathogens and environmental factors [5]. Considerable research has therefore focused on the pathophysiology of peripartum diseases and the interplay between metabolic imbalance and infection, often under experimental conditions designed to minimize environmental variability.

The inflammatory response associated with calving, ketosis, and mastitis is characterized by the systemic release of numerous cytokines and chemokines. Pro-inflammatory cytokines such as IL-1α/β, IL-6, and TNFα amplify inflammation, whereas IL-10, IL-1RA, and IL-4 contribute to its resolution [6]. Chemokines including CXCL8, CCL2, CCL3, CCL4 and CXCL10, recruit immune cells to inflammatory sites [6]. In cows, plasma cytokine profiling has revealed specific patterns, with multiplex assays of up to 15 cytokines distinguishing responses according to the pathogen type [7].

**Fig. 1:**
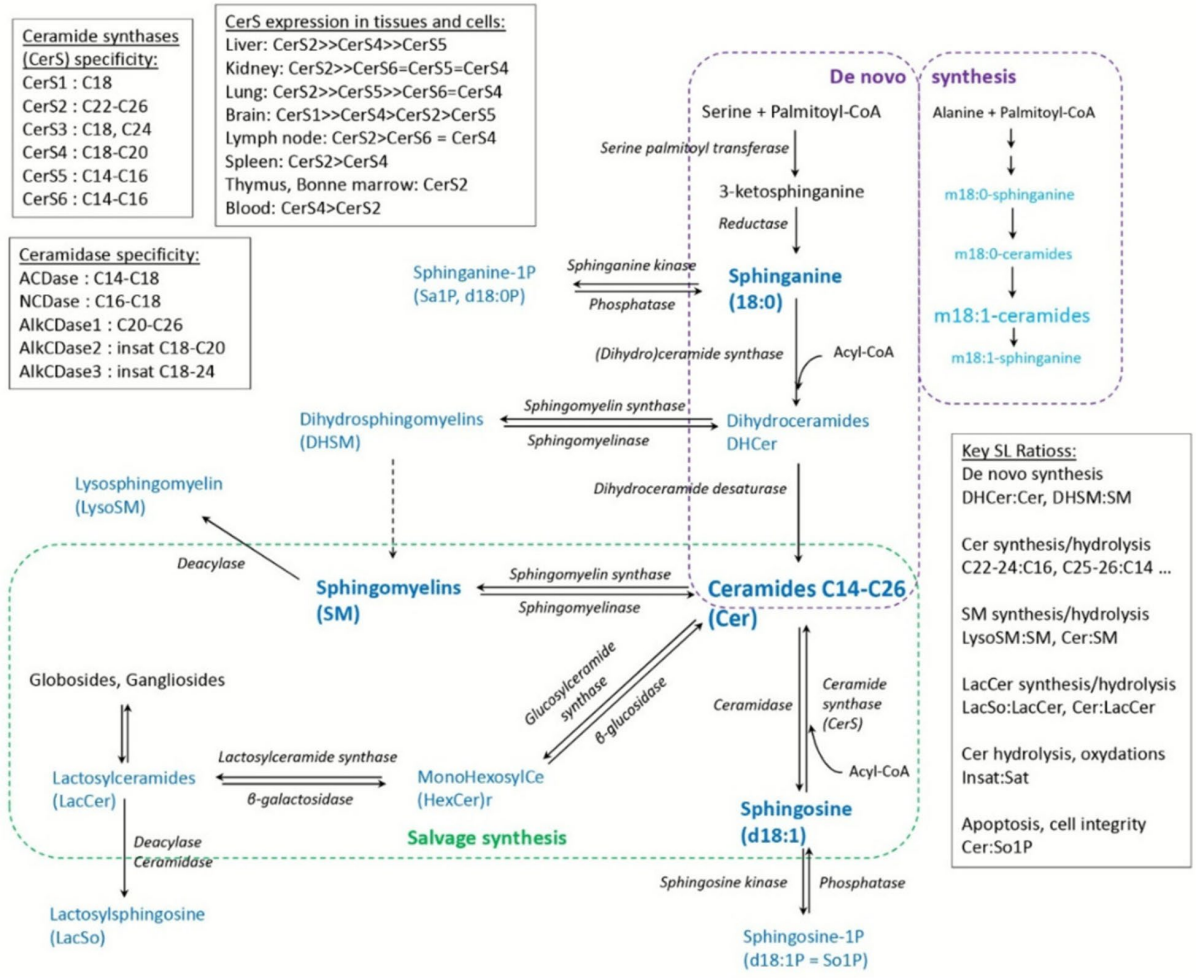
Simplified diagram of sphingolipid (SL) metabolic pathways and representative ratios for plasma sphingolipidome analysis

Sphingolipids (SL) are structurally diverse lipids that serve both membrane and signaling functions, playing central roles in the regulation of inflammation and immunity during infection and metabolic dysfunction [8, 9]. Major classes include sphingoid bases (SB), ceramides (Cer), sphingomyelins (SM), and glycosylceramides (GlyCer). Ceramides occupy a central position in SL metabolism (Fig. 1), arising from multiple pathways. In de novo synthesis, sphinganine is produced from serine and palmitoyl-CoA, then converted to dihydroceramides (DHCer), which are desaturated to Cer. Cer can be further modified to form monohexosylceramides (HexCer) and lactosylceramides (LacCer), or combined with phosphatidylcholine to generate SM. Dihydrosphingomyelins (DHSM) derive from DHCer. The salvage pathway regenerates Cer from hydrolyzed GlyCer and SM. Cer hydrolysis by ceramidases produces sphingosine, which can either be phosphorylated to sphingosine-1-phosphate (S1P) or re-acylated into Cer via ceramide synthases (CerS), isoenzymes with distinct tissue specificity [10–12]. Lysosphingolipids, such as lactosylsphingosine (LacSo) and lysosphingomyelin (LysoSM), represent deacylated derivatives.

Targeted SL profiling provides insights into the complex pathophysiological processes in which SL participate [8]. Specific SL ratios are frequently used as metabolic markers (Fig. 1). Elevated DHCer and DHSM, together with higher DHCer:Cer and DHSM:SM ratios, may indicate enhanced de novo synthesis [13]. Increased Cer:SM ratios suggest sphingomyelinase activation during inflammation [14]. Ratios between very long-chain (C22–24) and long-chain (C16) SL reflect shifts in CerS or ceramidase activity, with biological outcomes depending as much on chain-length distribution as on total concentration [12, 15, 16]. Elevated Cer and LacCer concentrations have been associated with pro-inflammatory cytokine synthesis and activation of phospholipase A2 (PLA2), leading to PUFA release and oxylipin production [17–19]. SM, conversely, may exert anti-inflammatory effects through membrane stabilization unless perturbed by hydrolysis or accumulation [17, 20]. Lysosphingolipid accumulation, reflected in LysoSM:SM or LacCer:Cer ratios, suggests ceramidase activation. Moreover, SL such as Cer, HexCer, LacCer, and SM contribute to the formation of membrane lipid rafts essential for pathogen recognition and attachment [21–23].

S1P is a particularly potent mediator, with its effects depending on receptor subtype. The Cer:S1P ratio functions as a rheostat controlling cell proliferation versus apoptosis [24]. Through S1PR3, S1P promotes differentiation of macrophages into pro-inflammatory phenotypes [25–27], while binding to S1PR1 favors anti-inflammatory macrophage polarization type [27–29]. Additional signaling roles through S1PR2, S1PR4, and S1PR5 encompass regulation of chemotaxis, vascular integrity, and immune cell trafficking [26, 30]. Finally, the ratio of saturated to unsaturated SL reflects both oxidative processes and modulation of ceramidase activity [11], with implications for inflammation in metabolic and infectious contexts. For example, acid ceramidase overexpression improves glucose metabolism and reduces insulin resistance in mice [31], whereas its inhibition during bacterial infection diminishes cytokine release and delays immune activation [32, 33].

Despite the central role of SL in inflammation, and even though both metabolic diseases and infections shape the postpartum inflammatory response, very limited information exists on sphingolipidomic alterations in cows. Most available studies have focused on ceramides in the context of ketosis [34, 35]. Relationships between lipid mobilization during the transition period and plasma and liver concentrations of Cer, HexCer, and LacCer, as well as between insulin resistance and certain Cer, have been suggested [36]. The role of SLs during the peripartum period in cows could therefore be similar to that described in obese humans and during the development of non-alcoholic fatty liver disease. Cer and S1P are key players in the development of diabetes and obesity in humans [37, 38]. An increase in de novo Cer synthesis is observed when a high-fat diet is administered in a mouse model of hepatic steatosis [39]. GlyCer are thought to play a major role in regulating inflammation and oxidative stress observed in experimental models of diabetes [40]. SL, in addition to being biomarkers of these conditions, appear to be tools for regulating them. In mice, targeted inhibition of serine palmitoyl transferase 2, a key enzyme in the regulation of de novo synthesis (Fig. 1), reduces hepatic and blood Cer levels and prevents lipogenesis, thereby attenuating the progression of hepatic steatosis [41, 42]. Inhibition of de novo Cer synthesis also prevents the production of pro-inflammatory cytokines [39]. Indeed, Cer, like LPS, binds to TLR4, leading to cytokine production and worsening inflammation [42, 43]. In humans and mice, pro-inflammatory cytokines, such as TNF-α and IL-1β, activate neutral sphingomyelinase, creating a feed-forward loop that exacerbates inflammation [44], whereas the translocation of neutral sphingomyelinase-2 to the plasma membrane drives insulin resistance in steatotic hepatocytes [45]. In a mouse model, gentiopicroside, a glycoside isolated from *Gentianaceae* [46], inhibits TLR4 and NLRP3 signaling pathways and prevents inflammation and hepatic fibrosis induced by pro-inflammatory macrophages [47]. However, little is known about SL-cytokine interactions in cows at postpartum.

The present study, therefore, aimed to characterize the impact of three major postpartum disorders—inflammation, ketosis, and mastitis—on plasma cytokine and SL profiles. The study was conducted in 80 Holstein cows within one week postpartum, selected from 427 animals across 25 farms. Four groups were defined to discriminate the specific effects of each condition. Cytokines were quantified using a multiplex assay enabling simultaneous measurement of 15 analytes [7], and plasma SL were profiled using targeted LC–MS/MS for detailed sphingolipidome characterization [48].

## Materials and methods

### Chemicals and reagents

The analytes and reagents used for sphingolipid extraction and quantification were obtained from Sharlab (Sharlab S.L., Sentmenat, Spain) and Sigma (Sigma-Aldrich Chimie SARL, Saint Quentin Fallavier, France). All reagents and solvents were of HPLC grade, except those used for sphingolipid extraction, which were LC–MS grade. Sphingolipid standards were purchased from Sigma. The internal standard (IS) mixture was Avanti Polar Lipids’ ‘Ceramide/Sphingoid Internal Standard (IS) Mixture I’, comprising C17-sphingosine, C17-sphinganine, C17-sphingosine-1 P, C17-sphinganine-1 P, C12:0-lactosyl(s)-ceramide, C12:0-sphingomyelin, C12:0-glucosyl(s)-ceramide, C12:0-ceramide, and C12:0-ceramide-1 P, supplemented with m17:1/12:0, m18:1/12:0, and C12:0-ceramide sulfatide.

Plasma haptoglobin levels (µg/mL) were quantified using a commercial ELISA kit (BIO K 328, Bio-X Diagnostics, France) following the manufacturer’s instructions. Plasma cytokines (pg/mL) were analyzed using a custom 15-plex bovine cytokine assay (SPRCUS617, Milliplex^®^ xMAP^®^, Merck-Millipore, France).

### Animal housing and sampling

All experimental procedures adhered to EU Directive 2010/63/EU and French regulations on animal care, and were approved by the Animal Ethics Committee, SSA N°115, under protocol SSA_2022_009. All breeders and project partners provided informed consent. The study monitored 427 Prim’Holstein cows from 25 farms in Brittany, France, over a four-month period (November 2023 to February 2024). All animals sampled in this study were parity 2 or 3. Complete sanitary data were available for 406 cows, which were included in the analysis [49].

Blood samples were collected on heparin 7 d post-calving, centrifuged at 2,500 × *g* for 15 min, and stored at −80 °C. Because calving alone can elevate haptoglobin to ~ 300 µg/mL at d 7 post-calving [50], a haptoglobin concentration above 400 µg/mL was considered pathological. Ketosis status was assessed using the “Cétodétect” indicator developed by Innoval (France), which estimates the risk of acetonemia from milk ketone measurements and zootechnical data [51]. Animals were scored from 0 to 5: scores of 0–1 indicated healthy cows, 2 indicated subclinical acetonemia, and 3–5 indicated clinical acetonemia. Milk cell counts were obtained from routine farm’s milk controls, retaining the value closest to calving. Counts exceeding 300,000 cells/mL were considered pathological [52, 53].

### Selection criteria and group composition

From the 427 cows monitored, 406 were selected based on complete sanitary status. Pathological cows included those showing clinical alterations such as metritis or lameness, or exhibiting plasma haptoglobin, ketosis score, or milk cell count values considered critical. Among the 406 cows, 243 were classified as controls and 163 as affected by pathology. The sphingolipidome analysis did not include cows with lameness, clinical metritis, or clinical mastitis.

**Fig. 2:**
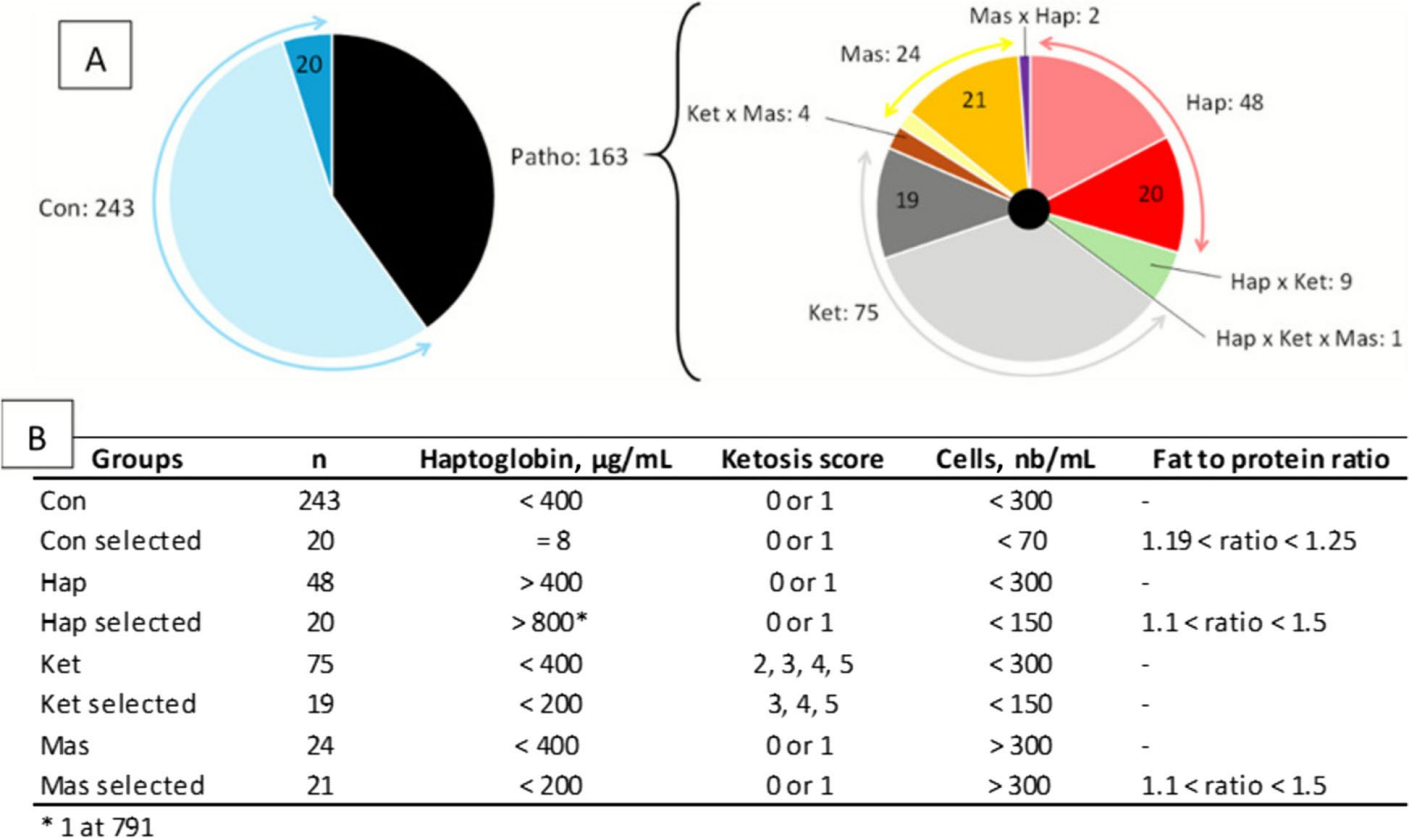
**A** Distribution of pathologies among the 406 lactating cows monitored. **B** The criteria used for forming the Con, Hap, Ket, and Mas groups

The Control group (Con) included cows with milk cell counts below 300,000 cells/mL, plasma haptoglobin below 400 µg/mL, and a ketosis score of 0 or 1. The Haptoglobin group (Hap) included cows meeting the control criteria except with haptoglobin above 400 µg/mL. The Ketosis group (Ket) included cows meeting the control criteria except with a ketosis score of 2–5. The Mastitis group (Mas) included cows meeting the control criteria except with milk cell counts above 300,000 cells/mL (Fig. 2A). For each group, 19–21 cows were selected to represent the most extreme profiles (Fig. 2B). Table S1 provides detailed values of the variables measured in the cows used in this study.

### Cytokine analysis

Plasma cytokine analysis was performed as described previously [7] using a custom 15-plex bovine cytokine assay (SPRCUS617, Milliplex^®^ xMAP^®^, Merck-Millipore, France). The analytes included five innate response cytokines (IL-1α, IL-1β, IL-1RA, IL-6, TNF-α), five adaptive response cytokines (IL-2, IL-4, IL-10, IFN-γ, IL-17A), and five chemokines (CCL2/MCP-1, CCL3/MIP-1α, CCL4/MIP-1β, CXCL8/IL-8, CXCL10/IP-10). Fluorescence intensities were measured on a MAGPIX^®^ system (Luminex^®^) equipped with a CCD camera and processed using xPONENT^®^ software (v4.2.1324.0, Luminex Corp., Austin, TX, USA). Cytokine concentrations (pg/mL) were calculated using calibration curves provided with the assay.

### UHPLC–MS/MS for sphingolipids

Sphingolipid extraction from plasma was performed as previously described [48, 54]. Briefly, 120 µL of plasma was diluted with 40 µL of 0.9% NaCl and spiked with 10 µL of an internal standard (IS) solution to achieve a final concentration of 6,250 pmol/mL plasma for each IS. Subsequently, 600 µL of methanol/chloroform (2:1, v/v) was added. Samples were incubated overnight at 48 °C. To remove potentially interfering glycerophospholipids [55], 100 µL of 1 mol/L KOH in methanol was added and incubated for 2 h at 37 °C, followed by the addition of 10 µL of 50% acetic acid. Samples were centrifuged, the supernatant collected, and the residue extracted again. The two supernatants were pooled, evaporated to dryness, and resuspended in 200 µL of methanol. After filtration, 10 µL was injected into the UHPLC system. Separation was performed on a Poroshell 120 EC18 column (3.0 mm × 50 mm, 2.7 µm, Agilent) using an Agilent 1260 autosampler and binary pump at 0.3 mL/min. Detection was performed in dynamic MRM mode on an Agilent 6410 triple quadrupole mass spectrometer with positive electrospray ionization at 300 °C, gas flow 10 L/min, and capillary voltage 4,000 V under 25 psi. Analytes corresponded to those listed in Table S2. Chromatograms were analyzed using Agilent MassHunter Quantitative Analysis software (B.05.291.0) with quadratic regression and 1/*x*^2^ weighting. Linearity was confirmed across a wide concentration range (Table S3). Precision, expressed as relative standard deviation (RSD), was < 20% and intra-day repeatability, assessed by IS recovery, is detailed in Table S4. For sphingolipids lacking commercial standards, concentrations were estimated using calibration curves from structurally similar analytes within the same class. Final plasma concentrations were corrected using the corresponding IS recovery.

### Analysis strategies and statistics

**Fig. 3:**
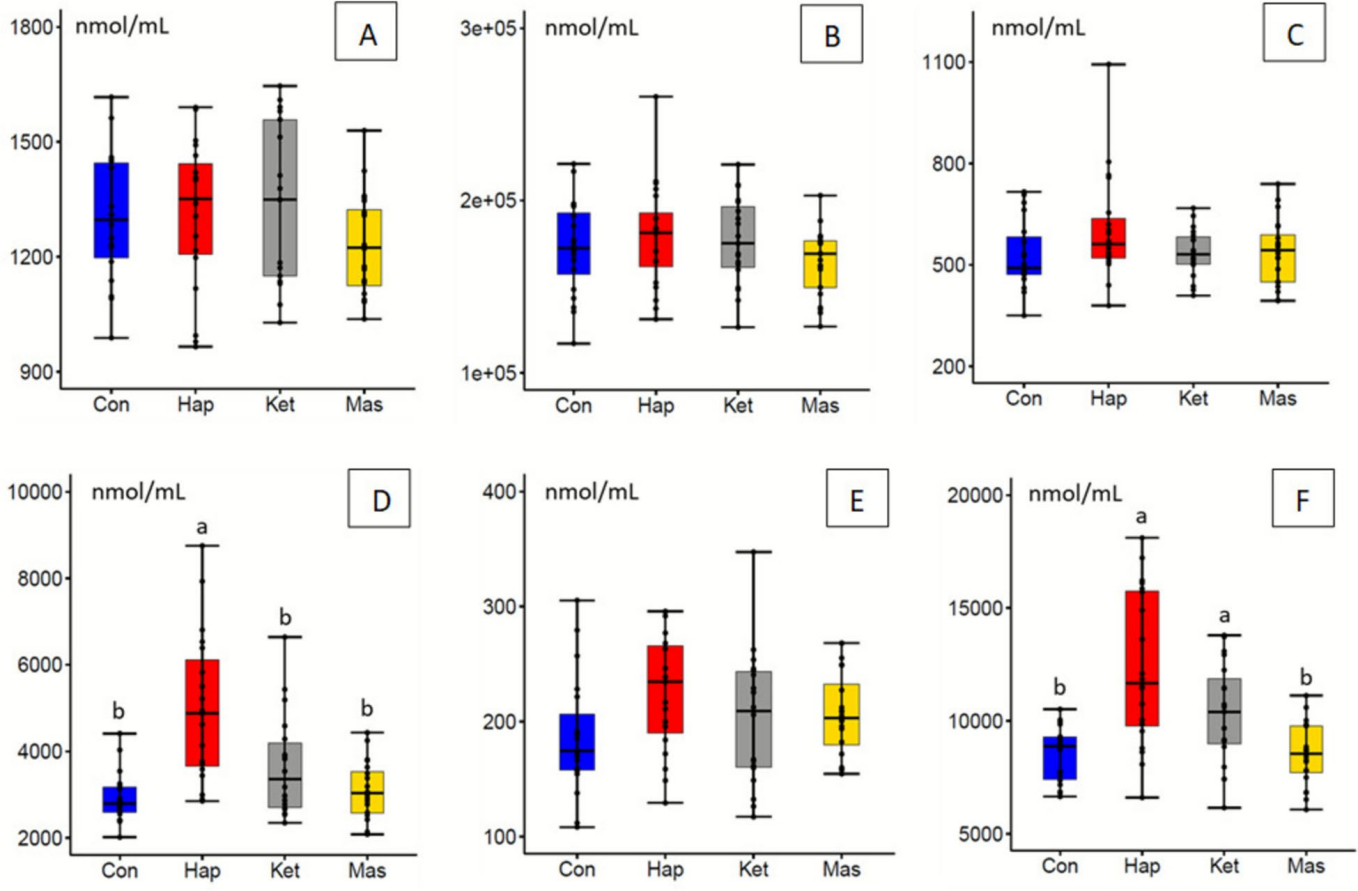
Plasma sphingolipid concentrations 7 d after parturition in different cow groups. Total plasma concentrations of **A** ceramides (Cer), **B** sphingomyelins (SM), **C** monohexosylceramides (HexCer), **D** lactosylceramides (LacCer), **E** dihydroceramides (DHCer), and **F** dihydrosphingomyelins (DHSM) were measured in control cows (Con), cows with hyperhaptoglobinemia (> 400 μg/mL, Hap), cows with ketosis score ≥3 (Ket), and cows with milk cell count > 300,000 cells/mL (Mas). Results are presented as mean ± SE, *n* = 19–1 per group. ANOVA followed by SNK post hoc test was used to assess differences between groups. Statistically different groups are indicated by different letters (*P* < 0.05)

Data are presented as mean ± SD in the Tables, while Figures display error bars as SE for improved clarity. All statistical analyses were performed using XLSTAT Biomed software (Addinsoft, Bordeaux, France) and R 4.3.0 (www.r-project.org). To reduce intra-group variability (visible in Fig. 3), all data were normalized based on the sum of total sphingolipids.

Initially, the impact of age, parity, and body condition score (BCS) of the cows, as well as the effects of systemic inflammation, ketosis, and mastitis on cytokine and sphingolipid concentrations, were assessed by ANOVA. Since many of the variables did not follow a normal distribution (Shapiro, *P* > 0.05), the analysis was performed on the log-transformed data. When significant differences among groups were observed (*P* < 0.05), the Student–Newman–Keuls (SNK) post hoc test was applied to assess differences between groups (*P* < 0.05). A small, significant effect of parity on IL-8, CCL-2, and CCL-3 levels was observed. No significant effects of age, BCS, systemic inflammation, ketosis, or mastitis on cytokines were found (Table S5). A significant effect of age was observed for five sphingolipids, primarily concerning 7-year-old cows (Table S6). A significant effect of parity was observed for one sphingolipid, while BCS significantly impacted two sphingolipids (Table S5). In contrast, systemic inflammation, ketosis, and mastitis significantly impacted 26 sphingolipids. Further analysis of interactions between the effects of age, parity, BCS, systemic inflammation, ketosis, and mastitis on sphingolipids was performed using two- or three-factor ANOVA. Between factors interaction that were significant have been reported in Table S6, most of them concerned the group of 7-year-old cows, which consisted of only two animals. Overall, these results show that the effects of age, parity, and BCS on the sphingolipidome are minor compared to the effects of systemic inflammation, ketosis, and mastitis. Consequently, the rest of the analysis focused on the effect of pathologies.

Partial least squares discriminant analysis (PLS-DA) was performed to investigate how systemic inflammation, ketosis, and mastitis impact cytokine and sphingolipid concentrations. However, no clear discrimination between the four groups was observed. Consequently, the effects of each health condition were compared in pairs with the control group. These PLS-DA analyses identified the variables most important in the projection (VIP) for group composition. A model was considered robust when Q^2^ > 0.5. If the model was robust but R^2^X was low, the number of analytes was reduced to improve model quality. This approach enhances parsimony and interpretability, typically by selecting analytes with the highest VIP values; in this study, VIP > 1.1 was used as the threshold.

Univariate analyses were subsequently performed to quantify differences between each pathology group and the control group. Normality was assessed using the Shapiro test for all analytes. For normally distributed analytes, ANOVA followed by the SNK post hoc test was applied. For non-normal analytes, Grubbs’ test was used to identify outliers (Fig. S1). The main assumption underlying this test is that abnormally high or low responses from one or two animals in a group could bias the interpretation of the results. These extreme values may also be due to analytical problems. If removal of extreme values restored normality, ANOVA with SNK post hoc testing was applied; otherwise, the non-parametric Kruskal–Wallis test was used (Fig. S2). Importantly, Grubbs’ test indicated that extreme values were not specific to individual cows, meaning that outlier removal affected specific analyte values rather than entire animals. Data obtained for all animals, without excluding outliers, are presented in the supplementary files (Tables S7–S9).

Ratios between sphingolipids were calculated to explore potential effects on specific synthesis pathways, and statistical analyses for ratios followed the same approach as for raw data (Fig. S2). Finally, Pearson correlation analyses were conducted between sphingolipids and cytokines within each group to identify potential interactions. Differences were considered significant at *P* < 0.05, and significance levels were indicated as * for 0.01 < *P* < 0.05, ** for 0.001 < *P* ≤ 0.01, and *** for *P* ≤ 0.001, or by different letters.

## Results

Haptoglobin concentration, ketosis score, and milk somatic cell count in the studied cows are presented in Fig. 2. Among the 406 cows with complete health and performance data, 163 were classified as affected; among which 48 exhibited only elevated haptoglobin (> 400 µg/mL), 75 showed only a ketosis score ≥ 2, and 24 displayed only a milk somatic cell count above 300,000 cells/mL. In addition, 9 cows presented both high haptoglobin and ketosis, 4 presented both ketosis and mastitis, 2 presented both mastitis and high haptoglobin, and 1 cow exhibited all three conditions.

### Overall impact of the health status on cytokines and sphingolipids

Partial least squares discriminant analysis (PLS-DA) performed on plasma cytokine concentrations across the four groups, or in pairwise comparisons between the control group (Con) and the other groups, failed to generate robust separation between animals. Similarly, ANOVA revealed no significant effects of the disease condition on cytokine concentrations (Table S7). Consequently, subsequent analyses focused on SL.

A PLS-DA performed on SL concentrations across all cows achieved 70% correct classification of animals into their respective groups (Fig. S3B). However, the robustness of the model was weak, with a Q^2^ value of 0.134 for the second component. After excluding SL with extreme values (Grubbs test), correct classification improved to 83%, though the Q^2^ value remained low (0.215; Fig. S3D). In agreement with this, no significant differences were observed in total plasma SL or in the total concentrations of Cer, SM, DHCer, or HexCer (Fig. 3). By contrast, significant differences were observed in DHSM and LacCer between the Con and Hap groups. Moreover, numerous individual SL species showed significant differences between groups according to ANOVA (Table 1). As some effects of the diseases were shared and others distinct, each one was subsequently analyzed separately using both PLS-DA and ANOVA.

**Table 1:** Effect of haptoglobin, ketosis, and mastitis on plasma sphingolipid concentrations

Variable	Control	Haptoglobin	Ketosis	Mastitis	*P*-value
m18:1	0.33 ± 0.01	0.35 ± 0.02	0.34 ± 0.01	0.38 ± 0.01	0.083
d18:1	51 ± 2.7	52.6 ± 3.9	54.1 ± 2.9	55.4 ± 2.5	0.671
d18:0	15.3 ± 0.6	14.7 ± 0.7	16 ± 0.8	16.3 ± 0.7	0.416
d18:1P	336 ± 13	363 ± 27	416 ± 33	343 ± 20	0.212
LacSo	16.8 ± 1^a^	13.1 ± 0.7^c^	13.8 ± 0.7^bc^	15.9 ± 0.7^ab^	0.003
LysoSM	46.3 ± 0.9^a^	39.8 ± 0.5^c^	42.4 ± 0.8^bc^	44.2 ± 0.7^b^	< 0.0001
Ceramides (Cer)					
18:1/14:0	4.51 ± 0.23^a^	2.96 ± 0.1^c^	3.78 ± 0.15^bc^	4.68 ± 0.21^ab^	< 0.0001
18:1/15:0	24.3 ± 1.1	20.9 ± 1	24.6 ± 1.1	24.2 ± 1.1	0.059
18:1/16:0	376 ± 13^a^	330 ± 12^b^	379 ± 13^a^	377 ± 16^a^	0.038
18:1/17:0	17.8 ± 0.5^a^	15.5 ± 0.5^b^	17.4 ± 0.4^a^	18 ± 0.5^a^	0.001
18:1/18:1	44.7 ± 1.1^a^	37 ± 1^b^	44.7 ± 1.2^a^	43.3 ± 1.4^a^	< 0.0001
18:1/18:0	42 ± 1.6	45.6 ± 2.8	45.4 ± 2.7	42.7 ± 1.3	0.880
18:1/20:0	9.25 ± 0.3	9.21 ± 0.46	10.16 ± 0.51	9.8 ± 0.33	0.332
18:1/22:0	94 ± 2	97 ± 3	101 ± 2	97 ± 2	0.303
18:1/23:0	114 ± 4	122 ± 6	114 ± 5	114 ± 5	0.687
18:1/24:2	115 ± 3^a^	103 ± 3^b^	117 ± 2^a^	119 ± 3^a^	0.001
18:1/24:1	170 ± 4	174 ± 6	175 ± 3	171 ± 4	0.801
18:1/24:0	133 ± 5	149 ± 7	132 ± 4	131 ± 4	0.130
18:1/25:1	116 ± 3^a^	103 ± 3^b^	116 ± 3^a^	120 ± 3^a^	0.001
18:1/25:0	26.5 ± 0.5	25.2 ± 0.7	27.1 ± 0.5	26.8 ± 0.7	0.130
18:1/26:0	24.7 ± 0.6^a^	22.9 ± 0.6^b^	25 ± 0.5^a^	24.4 ± 0.6^ab^	0.042
Dihydroceramides (DHCer)					
18:0/16:0	52.3 ± 3.5^ab^	65.3 ± 5.9^a^	47.4 ± 4^b^	68.4 ± 6.4^a^	0.022
18:0/17:0	4.98 ± 0.32^a^	5.06 ± 0.26^a^	4.23 ± 0.45^b^	5.73 ± 0.34^a^	0.008
18:0/18:0	12.5 ± 1.3^b^	18 ± 1.6^a^	19.3 ± 3.1^a^	17.6 ± 2^a^	0.029
18:0/20:0	1.73 ± 0.18	1.86 ± 0.2	2.56 ± 0.35	2.13 ± 0.16	0.191
18:0/22:0	15.7 ± 1.3^ab^	14.3 ± 1.2^b^	19.7 ± 2^a^	17.5 ± 1.3^ab^	0.046
18:0/23:0	50.5 ± 3.5	46.2 ± 3.3	54.1 ± 3.1	56.5 ± 2.4	0.066
18:0/24:0	54.2 ± 3.6	50 ± 2.9	57.9 ± 3.2	59 ± 3	0.117
Sphingomyelins (SM)					
SM18:1/14:0	2,350 ± 41^a^	2,075 ± 52^b^	2,356 ± 54^a^	2,421 ± 39^a^	< 0.0001
SM18:1/15:0	10,492 ± 117^a^	9,735 ± 168^b^	9,920 ± 113^b^	10,422 ± 159^a^	0.001
SM18:1/16:1	10,244 ± 196^a^	8,324 ± 313^b^	9,831 ± 255^a^	10,215 ± 219^a^	< 0.0001
SM18:1/16:0	26,498 ± 331^ab^	25,588 ± 456^b^	26,994 ± 462^a^	27,015 ± 228^a^	0.028
SM18:1/17:0	5,960 ± 144^a^	5,238 ± 178^b^	5,176 ± 137^b^	6,214 ± 187^a^	< 0.0001
SM18:1/18:2	155 ± 6^a^	133 ± 5^b^	143 ± 6^ab^	153 ± 8^a^	0.041
SM18:1/18:1	2,242 ± 34^bc^	2,148 ± 51^c^	2,293 ± 63^ab^	2,390 ± 35^a^	0.004
SM18:1/18:0	28,187 ± 309	28,370 ± 303	28,608 ± 377	28,156 ± 289	0.776
SM18:1/20:2	43.2 ± 1.2^bc^	40.5 ± 0.8^b^	41.7 ± 1.3^b^	47.6 ± 1.1^a^	0.000
SM18:1/20:0	3,206 ± 52	3,289 ± 45	3,317 ± 61	3,355 ± 37	0.191
SM18:1/22:2	359 ± 12	348 ± 11	351 ± 6	362 ± 7	0.675
SM18:1/22:1	4,294 ± 59^a^	3,922 ± 70^b^	4,206 ± 82^a^	4,214 ± 56^a^	0.001
SM18:1/22:0	44,179 ± 846	44,070 ± 722	43,796 ± 1,149	42,313 ± 586	0.410
SM18:1/23:1	7,205 ± 107^a^	6,262 ± 154^b^	6,804 ± 153^a^	7,163 ± 127^a^	< 0.0001
SM18:1/23:0	11,124 ± 79	11,199 ± 183	11,293 ± 171	10,908 ± 95	0.124
SM18:1/24:3	455 ± 21	418 ± 16	414 ± 18	478 ± 19	0.059
SM18:1/24:2	5,071 ± 106	4,675 ± 79	4,912 ± 120	5,022 ± 96	0.052
SM18:1/24:1	9,153 ± 227^bc^	9,809 ± 244^a^	9,016 ± 290^b^	8,735 ± 126^b^	0.011
SM18:1/24:0	8,626 ± 85	8,769 ± 111	8,689 ± 111	8,630 ± 89	0.751
SM18:1/25:2	321 ± 10^a^	283 ± 12^b^	284 ± 12^b^	350 ± 18^a^	0.003
SM18:1/25:1	848 ± 37	849 ± 45	764 ± 38	885 ± 46	0.237
SM18:1/25:0	1,633 ± 49^bc^	1,956 ± 84^a^	1,591 ± 85^c^	1,850 ± 74^ab^	0.003
SM18:1/26:3	60.6 ± 2.7^ab^	56.5 ± 1.5^b^	57.1 ± 2.4^b^	67.6 ± 2.9^a^	0.015
SM18:1/26:2	197 ± 7	198 ± 7	190 ± 11	215 ± 8	0.181
SM18:1/26:0	428 ± 20^b^	580 ± 35^a^	425 ± 26^b^	534 ± 31^a^	0.000
Dihydrosphingomyelins (DHSM)					
SM18:0/16:0	6,461 ± 126^c^	8,434 ± 297^a^	7,526 ± 198^b^	6,632 ± 130^c^	< 0.0001
SM18:0/18:0	837 ± 23^c^	1,093 ± 41^a^	943 ± 28^b^	948 ± 35^b^	< 0.0001
SM18:0/20:0	387 ± 12^b^	564 ± 28^a^	429 ± 14^b^	426 ± 13^b^	< 0.0001
SM18:0/22:0	383 ± 22^b^	496 ± 23^a^	403 ± 21^b^	422 ± 20^b^	0.004
SM18:0/23:0	215 ± 6^c^	320 ± 15^a^	250 ± 10^b^	235 ± 9^bc^	< 0.0001
SM18:0/24:1	104 ± 6^b^	200 ± 13^a^	120 ± 7^b^	111 ± 6^b^	< 0.0001
SM18:0/24:0	145 ± 4^b^	215 ± 8^a^	153 ± 3^b^	156 ± 7^b^	< 0.0001
Monohexosylceramides (HexCer)					
Hex18:1/16:0	64.1 ± 3^b^	78.7 ± 4.1^a^	64.8 ± 1.9^b^	67.3 ± 2.4^b^	0.008
Hex18:1/18:0	22.5 ± 1.4	24.4 ± 1.5	22.7 ± 0.8	22.7 ± 1	0.711
Hex18:1/22:0	209 ± 10	216 ± 12	201 ± 6	212 ± 5	0.746
Hex18:1/24:1	154 ± 9	166 ± 9	142 ± 6	161 ± 6	0.154
Hex18:1/24:0	100 ± 5	106 ± 5	104 ± 4	109 ± 4	0.444
Lactosylceramides (LacCer)					
Lac18:1/16:0	2,600 ± 71^b^	4,114 ± 187^a^	2,993 ± 128^b^	2,906 ± 152^b^	< 0.0001
Lac18:1/18:0	59.4 ± 3.7	70.2 ± 4.5	67.5 ± 4.6	62.9 ± 1.7	0.255
Lac18:1/22:0	94 ± 4	101 ± 5	111 ± 5	101 ± 4	0.116
Lac18:1/24:1	118 ± 6^b^	164 ± 18^a^	137 ± 8^ab^	126 ± 8^b^	0.037
Lac18:1/24:0	72.2 ± 2.6^b^	75.5 ± 2.9^b^	85.8 ± 1.9^a^	78.1 ± 2.6^b^	0.003

Values are expressed in nmol/mL of plasma as mean ± SE. Sample sizes: *n* = 20 for control and haptoglobin groups, *n* = 19 for ketosis group, and *n* = 21 for mastitis group. Grubbs tests were performed to remove extreme values (Table S5). ANOVA was used when the analyte followed a normal distribution; otherwise, Kruskal–Wallis was applied (values underlined). Statistically different groups are indicated by different letters (*P* < 0.05)

### Effect of inflammatory condition on the plasma sphingolipidome

**Fig. 4:**
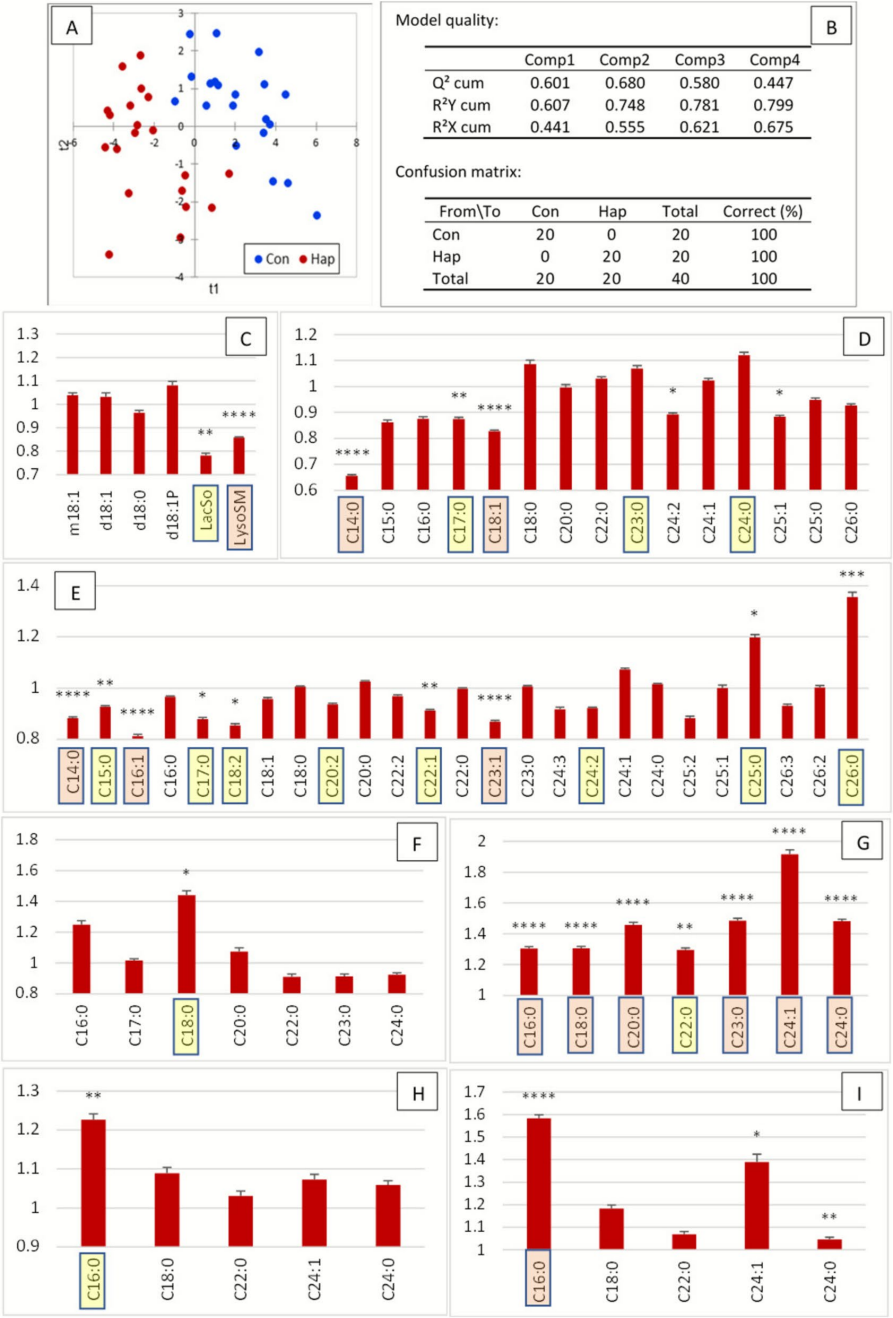
Plasma sphingolipid profile 7 d after parturition in control cows (Con) and cows with hyperhaptoglobinemia (> 400 µg/mL, Hap). **A** Partial least squares discriminant analysis (PLS-DA) showing group separation on the factor axes derived from the original explanatory variables. **B** Model quality metrics and confusion matrix. **C**–**I** Plasma sphingolipid concentrations expressed as fold change relative to the control group (mean ± SE, *n* = 20 per group) for **C** sphingoid bases and derivatives, **D** ceramides, **E** sphingomyelins, **F** dihydroceramides, **G** dihydrosphingomyelins, **H** monohexosylceramides, and **I** lactosylceramides. Variables important in the projection (VIP) for the first and second components are highlighted according by color: yellow, 1.1 < VIP < 1.4; red, VIP > 1.4. Statistically significant differences between groups (ANOVA) are indicated as: * 0.05 < *P* < 0.01, ** 0.01 < *P* < 0.001, *** *P* < 0.0001

PLS-DA achieved excellent separation between Con and Hap cows, with 100% specificity and 100% accuracy, supported by a robust Q^2^ value of 0.68 (Fig. 4A and B). Variables important for projection (VIP) are presented in Fig. S4 and Fig. 4C–E. ANOVA identified 28 SL species that differed significantly between the two groups (Fig. 4C–E).

Sphingoid bases (SB) did not differ between groups, while LacSo and LysoSM were significantly reduced in Hap cows compared with controls (Fig. 4C). Several Cer species were decreased in Hap cows: among saturated Cer, the strongest decreases were observed for 18:1/14:0 and 18:1/17:0 (Fig. 4D), while among unsaturated Cer, the most affected were 18:1/18:1, 18:1/24:2, and 18:1/25:1. In contrast, 18:1/18:0, 18:1/24:0, and 18:1/25:0 remained unchanged.

SM were more strongly affected than Cer, with sharp decreases in C14:0, C15:0, and C17:0 species. Similarly, SM C16:1, C18:2, and C22:1 were decreased in Hap cows, whereas their saturated counterparts (C16:0, C18:0, C22:0) were unaffected (Fig. 4E). Only very long-chain SM species C25:0 and C26:0 were increased.

In contrast to the reduction in Cer and SM, dihydrosphingolipids (DHSL) were consistently increased in Hap cows, with more pronounced effects on DHSM than on DHCer. Indeed, only DHCer 18:1/18:0 was increased, whereas all DHSM species were elevated (Fig. 4F and G). GlyCer were also elevated in the Hap group. Among these, HexCer 18:1/16:0 was increased, while multiple LacCer species (C16:0, C24:1, C24:0) were significantly elevated (Fig. 4H and I).

The most discriminating VIP identified by PLS-DA corresponded to the SL species most significantly affected. Notably, all DHSM were identified as VIP, with six out of seven species exceeding a VIP value of 1.4 (Fig. 4G). Other key VIP included C14–C18 Cer and SM, very long-chain unsaturated Cer, and very long-chain saturated SM (Fig. 4D and E).

In summary, increased haptoglobinemia was associated with profound remodeling of the plasma sphingolipidome. While SB remained unchanged, lysosphingolipids were reduced, and both Cer and SM showed chain length- and saturation-specific decreases. Conversely, DHCer, DHSM, and GlyCer—particularly LacCer—were consistently elevated. The most strongly affected SL species were also those with the highest discriminating power in multivariate analysis.

### Effect of ketosis on the plasma sphingolipidome

**Fig. 5:**
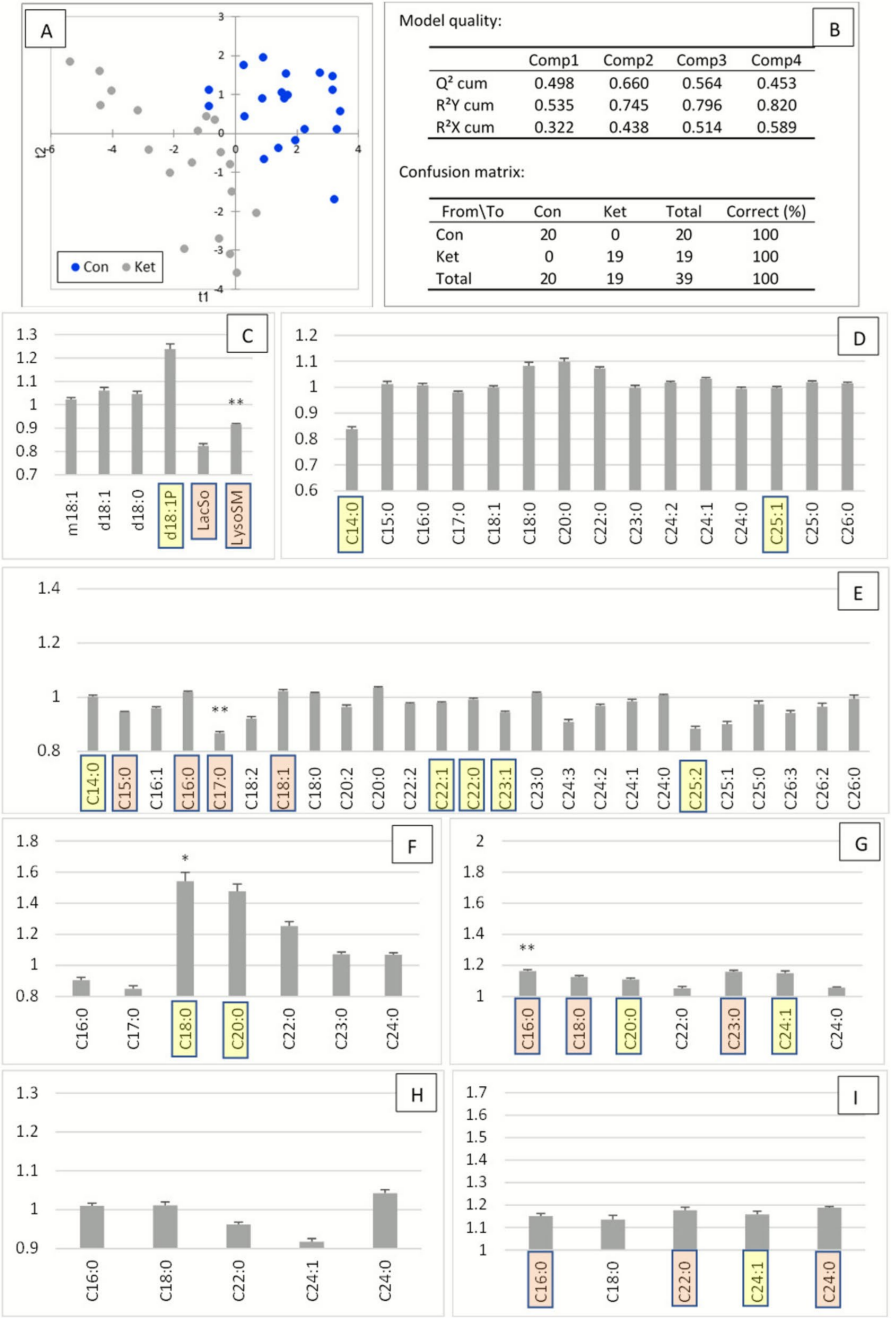
Plasma sphingolipid profile 7 d after parturition in control cows (Con) and cows with ketosis (Ket, score ≥ 3). **A** Partial least squares discriminant analysis (PLS-DA) showing separation between the two groups on the factor axes derived from the original explanatory variables. **B** Model quality metrics and confusion matrix. **C**–**I** Plasma sphingolipid concentrations expressed as fold change relative to the control group (mean ± SE; *n* = 20 for Con, *n* = 19 for Ket) for **C** sphingoid bases and derivatives, **D** ceramides, **E** sphingomyelins, **F** dihydroceramides, **G** dihydrosphingomyelins, **H** monohexosylceramides, and **I** lactosylceramides. Variables important in the projection (VIP) for the first and second components are highlighted according by color: yellow, 1.1 < VIP < 1.4; red, VIP > 1.4. Statistically significant differences between groups (ANOVA) are indicated as: * 0.05 < *P* < 0.01, ** 0.01 < *P* < 0.001, *** *P* < 0.0001

PLS-DA achieved excellent separation between Con and Ket cows, with 100% specificity and 100% accuracy, supported by a robust Q^2^ value of 0.66 (Fig. 5A and B). VIP values are presented in Fig. S5 and Fig. 5C–E. However, despite the strong multivariate discrimination, only four SL species were significantly different between groups according to ANOVA (Fig. 5C–E).

Among sphingoid bases, d18:1P was identified as a VIP and showed a slight increase in Ket cows relative to controls (Fig. 5C). LacSo and LysoSM were also important VIP, both decreased in the Ket group, although only the reduction in LysoSM reached significance (Fig. 5C). Cer were minimally affected: only 2 out of 15 species were VIP (both with VIP < 1.4), and no significant differences in Cer concentrations were detected (Fig. 5D).

SM showed more variable responses according to chain length. Nine SM species were VIP, with C15–C18 SM exceeding a VIP value of 1.4. SM18:1/14:0 and SM18:1/16:0 tended to increase, whereas SM18:1/15:0 and SM18:1/17:0 tended to decrease, the latter being significantly reduced compared to controls.

Among dihydrosphingolipids, both C18 and C20 DHCer were VIP and increased, although only DHCer 18:0/18:0 was significantly higher in Ket cows (Fig. 5F). Five of seven DHSM were VIP (three with VIP > 1.4), with SM18:0/16:0 being significantly elevated (Fig. 5G). Ketosis had no detectable effect on HexCer. By contrast, four of five LacCer species were VIP, three of them with VIP > 1.4, although none differed significantly in concentration (Fig. 5H and I).

Overall, this combined PLS-DA and ANOVA analysis showed that many of the SL most influential in the model (high VIP) did not show significant change in concentration between groups. The impact of ketosis on the plasma sphingolipidome was thus more qualitative than quantitative. Specifically, ketosis was associated with an increase in d18:1P and a decrease in lysosphingolipids, while Cer remained unchanged. SM exhibited chain length–dependent effects, whereas DHSM and LacCer tended to increase, many of them being among the most discriminating VIP.

### Effect of mastitis on the plasma sphingolipidome

**Fig. 6:**
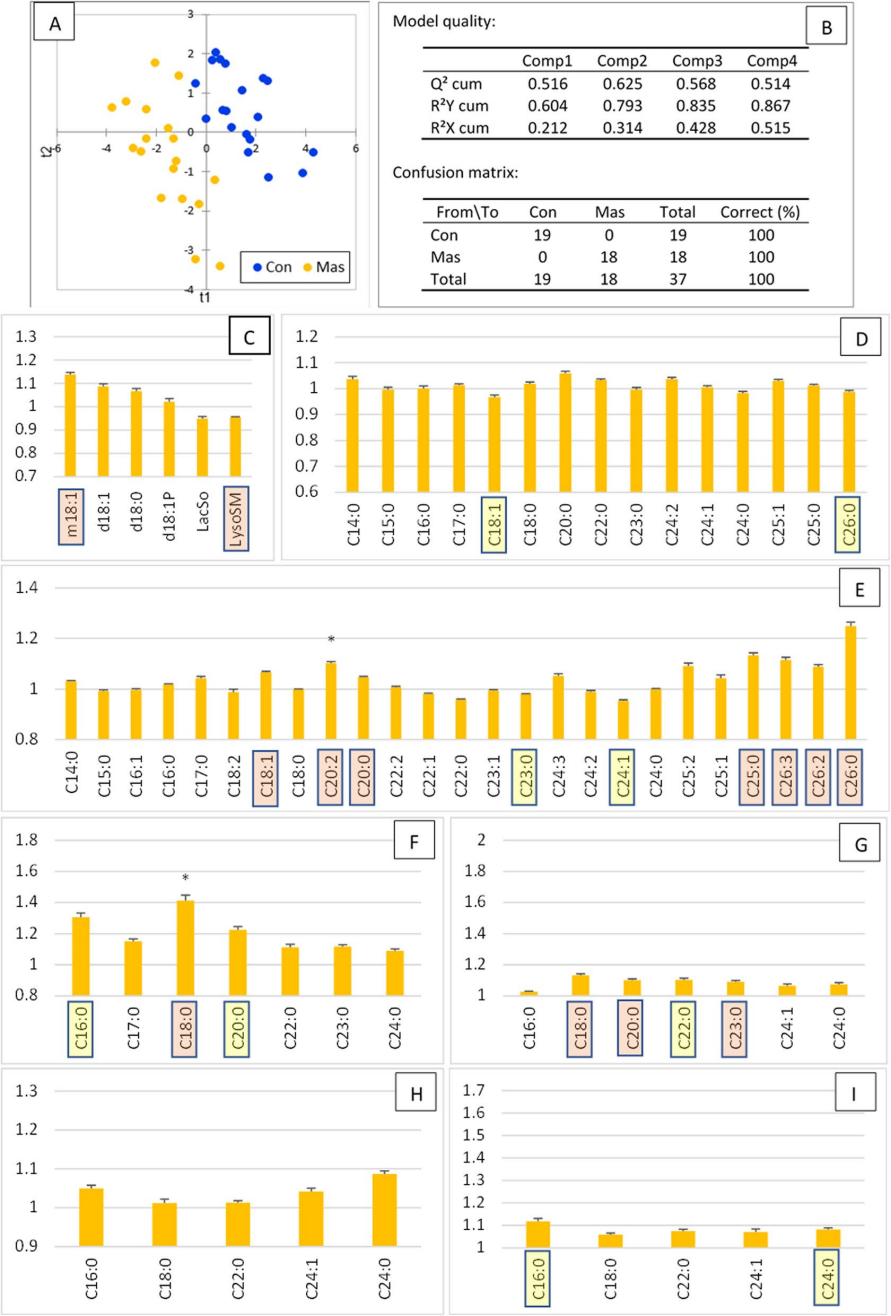
Plasma sphingolipid profile 7 d after parturition in control cows (Con) and cows with mastitis (Mas, milk cell count > 300,000 cells/mL). **A** Partial least squares discriminant analysis (PLS-DA) showing separation between the two groups on the factor axes derived from the original explanatory variables. **B** Model quality metrics and confusion matrix. **C**–**I** Plasma sphingolipid concentrations expressed as fold change relative to the control group (mean ± SE; *n* = 19 for Con, *n* = 18 for Mas), for **C** sphingoid bases and derivatives, **D** ceramides, **E** sphingomyelins, **F** dihydroceramides, **G** dihydrosphingomyelins, **H** monohexosylceramides, and **I** lactosylceramides. Variables important in the projection (VIP) for the first and second components are highlighted according by color: yellow, 1.1 < VIP < 1.4; red, VIP > 1.4. Statistically significant differences between groups (ANOVA) are indicated as: * 0.05 < *P* < 0.01, ** 0.01 < *P* < 0.001, *** *P* < 0.0001

An initial PLS-DA of the 20 Con and 21 Mas cows revealed some overlap: one control cow was misclassified, and three mastitis cows projected close to controls on the t1 and t2 axes (Fig. S6A and 6B). To better identify discriminating plasma SL, these four animals were excluded, after which the PLS-DA achieved excellent separation with 100% specificity and accuracy, supported by a robust Q^2^ value of 0.625 (Fig. 6A and B). VIP are shown in Fig. S6C–F and Fig. 6C–E. Although many SL species had VIP > 1.4, only three were significantly different between Con and Mas cows (Fig. 6C–E).

Among sphingoid bases, m18:1 was a strong VIP but did not differ significantly in concentration between groups. LysoSM, also an important VIP, was significantly reduced in mastitis cows compared with controls (Fig. 6C).

Cer were only marginally affected: two of fifteen species were VIP, neither with VIP > 1.4, and no significant group differences were observed (Fig. 6D). By contrast, SM showed stronger discrimination: 9 of 25 SM were VIP, 7 with values > 1.4. Notably, all C26 SM species and SM18:1/25:0 exceeded the 1.4 threshold and tended to increase in mastitis cows, although only SM18:1/20:2 was significantly higher (Fig. 6E).

Dihydrosphingolipids were also moderately affected. Three of seven DHCer and four of seven DHSM were VIP, all slightly elevated in mastitis cows compared with controls (Fig. 6F and G). The increase was stronger for DHCer, reaching significance for DHCer 18:0/18:0, while more DHSM exceeded the VIP > 1.4 threshold. Mastitis had no detectable effect on HexCer (Fig. 6H). Two LacCer species were identified as VIP, but none exceeded a value of 1.4, nor did their concentrations differ significantly between groups.

In summary, mastitis exerted only a modest quantitative impact on the plasma sphingolipidome. m18:1 showed a tendency to increase, while LysoSM was significantly reduced. Cer were largely unaffected, whereas very-long-chain SM species (notably C26 SM) tended to increase. Both DHCer and DHSM were slightly elevated, with stronger effects on DHCer, while LacCer were minimally influenced.

In conclusion, this study revealed that systemic inflammation identified through plasma haptoglobin concentration induced profound quantitative and qualitative alterations in the plasma sphingolipidome. By contrast, ketosis and mastitis exerted only limited quantitative effects, though PLS-DA clearly separated affected cows from controls, suggesting important qualitative remodeling of SL profiles. These findings prompted the subsequent analysis of SL ratios, aimed at identifying alterations in biosynthetic pathways specific to each pathology (Fig. 1).

### Characterization of sphingolipid ratios

SL ratios were calculated to identify the biosynthetic and catabolic pathways most affected in the different categories. At the sphingoid base (SB) level, neither the m18:1/d18:1 nor the d18:1P/d18:1 ratio differed significantly among groups (Table S9). Similarly, the So1P:Cer ratio was unchanged.

**Fig. 7:**
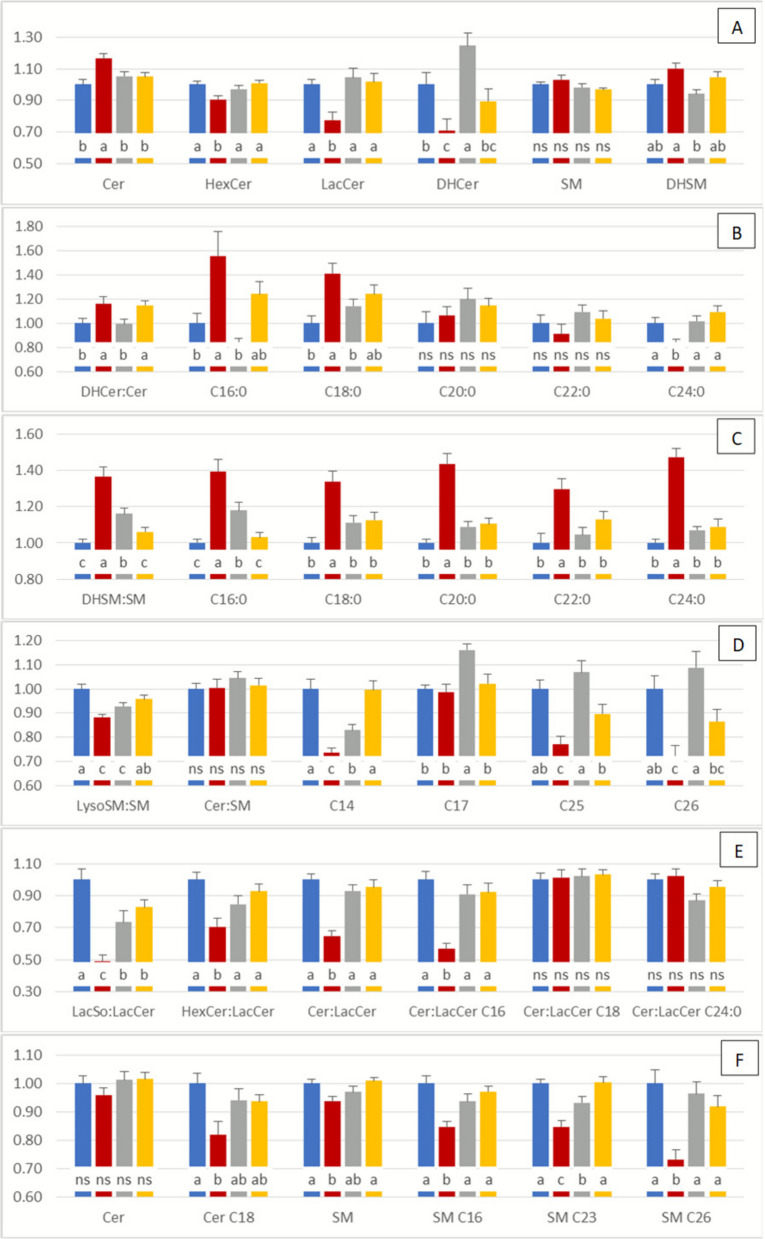
Plasma sphingolipid (SL) ratios 7 d after parturition in control cows (Con), cows with haptoglobinemia (Hap, haptoglobin > 400 µg/mL), cows with ketosis (Ket, score ≥ 3), and cows with mastitis (Mas, milk cell count > 300,000 cells/mL). **A** Ratios of C22-24:C16 measured across different SL classes. **B** DHCer:Cer ratios, **C** DHSM:SM ratios, **D** LysoSM:SM and Cer:SM ratios, **E** LacSo:Cer, HexCer:LacCer and LacCer:Cer ratios, **F** insat:sat ratios for Cer and SM. Values are expressed as mean ± SE (*n* = 20 for Con and Hap, *n* = 19 for Ket, *n* = 21 for Mas). Differences among groups were assessed using ANOVA, and statistically different groups (SNK) are indicated by different letters (*P* < 0.05)

Ratios comparing SL of different chain lengths are informative about ceramide synthase (CerS) and ceramidase activity (Fig. 1). In Hap cows, the C22-24:C16 ratio increased in Cer, but decreased in HexCer, LacCer, and DHCer (Fig. 7). By contrast, ketosis selectively increased the C22-24:C16 ratio in DHCer, while mastitis had no effect. Ratios in SM and DHSM did not differ from controls regardless of disease. Interestingly, the sharp decrease in 18:1/14:0 observed in Hap and Ket cows was mirrored by sharp increases in the ratios of all other Cer to this analyte (Table S9). Similarly, SM ratios relative to SM18:1/14:0 were strongly increased in Hap cows.

The DHCer:Cer and DHSM:SM ratios are indicative of de novo SL synthesis (Fig. 1). The DHCer:Cer ratio (sum of analytes) was increased in Hap and Mas cows, largely driven by effects on C16 and, to a lesser extent, C18 species (Fig. 7). Ratios for C20 and C22 species remained unaffected, while C24:0 decreased in Hap cows. The DHSM:SM ratio was increased in Hap and Ket groups: in Hap cows this reflected a consistent increase across C16–C24 species, whereas in Ket cows it was restricted to C16 (Fig. 7).

Ratios between complex SL can reveal changes in enzymatic activity or substrate flux (Fig. 1). The LysoSM:SM ratio decreased in Hap and Ket cows, whereas the overall Cer:SM ratio was unchanged (Fig. 7). However, specific decreases in Cer:SM were detected for low-abundance SL species (C14, C25, C26) in Hap cows and (C25, C26) in Mas cows, while an increase at C17 was observed in Ket cows. The low plasma abundance of these species likely masked these effects when analyzing total Cer:SM.

The LacSo:LacCer ratio was consistently reduced across all diseases, with the most pronounced decrease in Hap cows (Fig. 7A). Cer:LacCer and HexCer:LacCer ratios also decreased in Hap cows, mainly due to changes in C16 species, whereas other chain lengths were unaffected.

Finally, unsaturation ratios were examined. For Cer, the overall insat:sat ratio did not differ, but the C18 subset was significantly reduced in Hap cows (Fig. 7F). For SM, the overall insat:sat ratio decreased in Hap cows (Fig. 7F), driven by significant effects across C16, C18, C20, C23, C25, and C26 species (Fig. 7F, Table S9).

Overall, ratio analysis uncovered subtle but health condition-specific alterations in SL metabolism that were not apparent from concentration data alone.

### Correlations between cytokines and sphingolipids

**Fig. 8:**
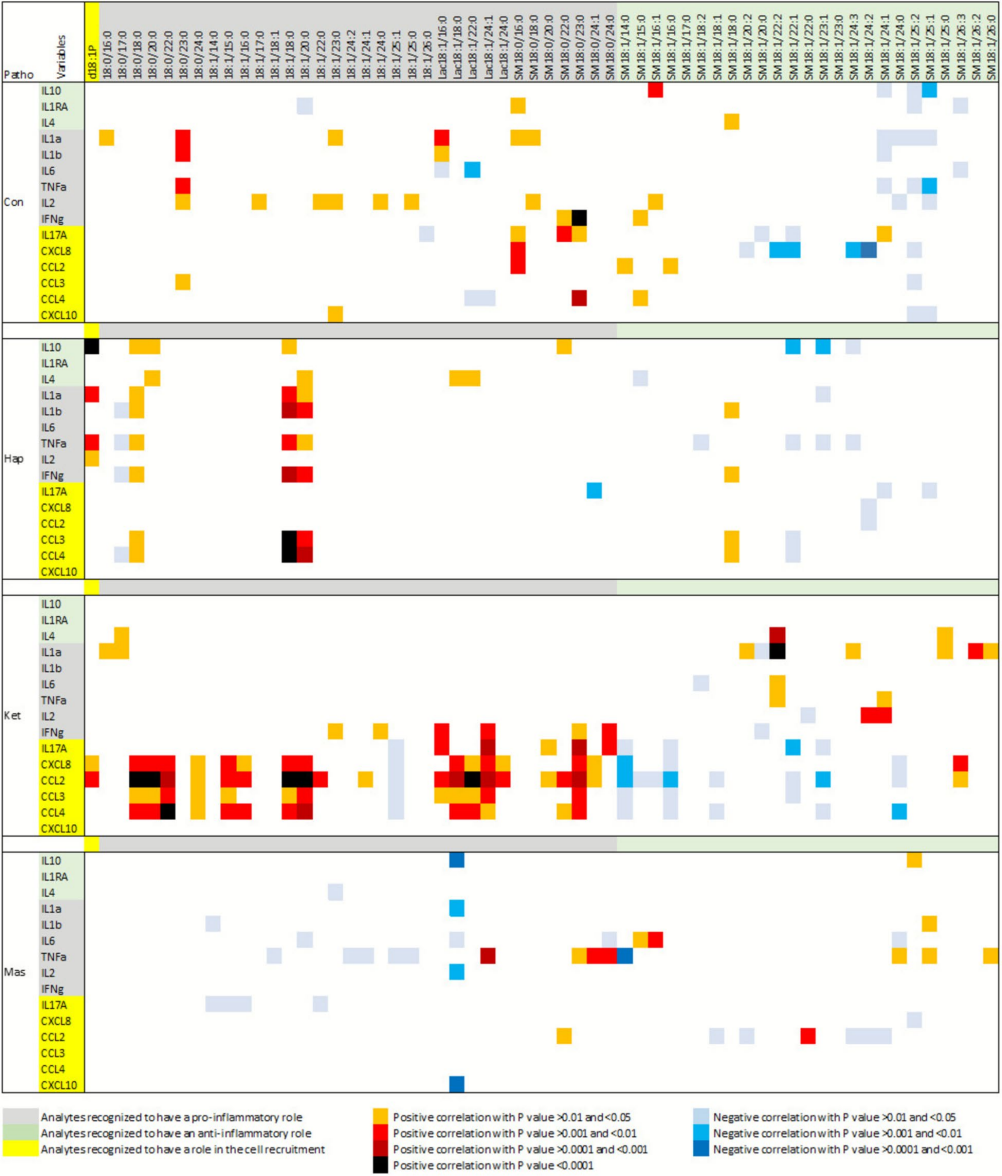
*P*-value of Pearson correlations between cytokines and sphingolipids found 7 d after parturition in the plasma of control cows (Con), cows with haptoglobinemia exceeding 400 µg/mL (Hap), cows with a ketosis score greater than or equal to 3 (Ket), and cows with milk cell count above 300,000 cells/mL (Mas). R value of correlation matrix are shown in Table S10

Because both SL and cytokines regulate inflammation, correlations between them were explored within each group (Fig. 8, Table S10).

#### Controls

In healthy cows, correlations were sparse and generally weak. Occasional positive associations were found between pro-inflammatory cytokines (IL-1α, IL-1β, IL-6, TNFα, IL-2, IFNγ) and Cer, DHCer, LacCer, or DHSM (Fig. 8A). Chemokine correlations were similarly limited, with the exception of a very strong positive correlation between IFNγ and SM18:0/23:0 (*R* = 0.784, *P* < 0.0001). Chain-length specificity was observed: SM at C15–C18 rarely correlated, while SM at C22–C26 showed more frequent and often negative correlations, particularly with CXCL8.

#### Elevated haptoglobinemia

In Hap cows, strong positive correlations were observed between d18:1P and IL-10, IL-1α, TNFα, and, to a lesser extent, IL-2 (Fig. 8B). Pro-inflammatory cytokines and chemokines also correlated positively with C18–C20 Cer. There was also a weaker correlation with C18 DHCer. Highly significant correlations were found between CCL3/CCL4 and 18:1/18:0 or 18:1/20:0. Conversely, correlations with SM were rare and mainly negative, except for positive associations of SM18:1/18:0 with cytokines, and significant correlations between IL-10 and SM18:1/22:1 and SM18:1/23:1.

#### Ketosis

In Ket cows, IL-10, IL-1RA, IL-1β, and CXCL10 showed no correlations with SL (Fig. 8C). Sporadic correlations were seen between SM and IL-4, IL-1α, IL-1β, IL-6, TNFα, and IL-2, including a strong correlation between SM18:1/22:2 and IL-1α (*R* = 0.803, *P* < 0.0001). By contrast, chemokines (CXCL8, CCL2, CCL3, CCL4) displayed numerous strong positive correlations with DHCer, Cer, LacCer, and DHSM, but generally negative correlations with SM. CCL2 was the most strongly associated chemokine, with particularly robust correlations to 18:0/18:0, 18:0/20:0, 18:1/18:0, and 18:1/20:0 (*P* < 0.0001). CCL4 was also strongly correlated with 18:0/22:0. LacCer displayed uniformly strong and positive correlations across species, without the chain-length specificity observed for Cer or DHCer. The strongest overall correlation was between CCL2 and Lac18:1/22:0 (*R* = 0.812, *P* < 0.0001).

#### Mastitis

In Mas cows, correlation patterns were less pronounced (Fig. 8D). Apart from a consistent negative association of Lac18:1/18:0 with five cytokines, and a positive correlation of TNFα with eight SL species, no major trends were observed (Table S10).

## Discussion

### Selection of animals

In this study, 48 cows out of 406 presented a blood concentration of haptoglobin above 400 µg/mL, the most frequent alteration observed, consistent with previous reports [2, 56]. Haptoglobin, a liver-derived glycoprotein migrating in the α2-globulin fraction, is a major acute-phase protein reflecting acute inflammation. Its post-partum dynamics vary widely between animals after calving [57, 58]. Higher concentrations are generally associated with more severe tissue damage of the genital tract after calving and increased occurrence of infection [2, 58, 59], with metritis identified as the predominant cause of hyperhaptoglobinemia [2, 58]. This condition is usually accompanied by systemic increases in IL-1β, IL-6, and TNF-α [2]. Because cows with clinical metritis or other overt diseases were excluded, the Hap group in this study likely represents animals experiencing acute but subclinical inflammation attributable to genital tract infection or another undetected cause.

Seventy-five cows exhibited subclinical and clinical ketosis (ketosis score ≥ 2), among them thirty-nine with clinical ketosis (ketosis score ≥ 3), according to the composite indicator based on MIR data and milk composition. Ketosis is a common and costly peripartum metabolic disease, impairing both performance and welfare [3]. It results from the mismatch between the sharp energy demand at lactation onset and insufficient feed intake, producing a negative energy balance, mobilization of non-esterified fatty acids, and accumulation of ketone bodies such as β-hydroxybutyrate. Among cows with a ketosis score > 2, nine also had elevated haptoglobinemia and four had SCC > 300,000/mL. This aligns with earlier studies showing that even subclinical ketosis may coincide with increased haptoglobinemia and systemic increases in IL-1β, IL-6, IL-8, and TNF-α [60]. Moreover, the coexistence of hyperketonemia and mastitis post-calving is well documented [56]. To isolate the metabolic effect of ketosis on the sphingolipidome, only cows with haptoglobinemia < 200 µg/mL and SCC < 150,000/mL were retained in the Ket group.

Thirty cows presented SCC > 300,000/mL, a widely recognized marker of mastitis which is one of the most important health issues in dairy farming worldwide [61, 62]. Within this cohort, 2 cows also showed haptoglobinemia > 400 µg/mL, while 24 had SCC > 300,000/mL with haptoglobinemia < 200 µg/mL, consistent with the variable acute phase response reported during a mastitis episode [2, 63]. For the Mas group, cows with SCC > 300,000/mL and haptoglobinemia < 200 µg/mL were selected, excluding one animal with a ketosis score > 3.

### Effect of diseases on cytokines

PLS-DA did not discriminate animals based on plasma cytokine profiles, and only weak correlations were detected between cytokine concentrations, haptoglobinemia, ketosis score, and SCC. This was unexpected, as several studies have reported significant associations between haptoglobinemia and cytokines during metritis [50, 64–66], ketosis and peripartum metabolic stress [60, 65, 67–69] and mastitis [59, 70–73]. IL-8 has even been proposed as an auxiliary biomarker for ketosis [74]. Furthermore, mastitis is consistently shown to raise cytokine concentrations in milk, and in severe forms, in plasma as well [70–81].

A key distinction, however, is that most studies reporting elevations of plasma cytokine concentrations were conducted under experimental challenge conditions, often with severe health outcomes and sometimes mortality. These contexts amplify inflammatory responses and their systemic signatures. In contrast, the present study was carried out under field conditions on cows without overt clinical disease. Under such circumstances, cytokine levels are influenced not only by subclinical conditions but also by multiple environmental and physiological factors. This complexity likely explains the absence of a clear group separation by PLS-DA.

### Impact of high haptoglobinemia on the sphingolipidome

High haptoglobinemia in dairy cows was associated with extensive alterations in the plasma SL. No direct comparisons can be made with previous bovine data, but the joint elevation of DHCer:Cer and DHSM:SM ratios, together with the overall increase in DHSM, strongly suggests enhanced de novo SL synthesis (Fig. 1). This phenomenon is consistent with the acute inflammatory response that characterizes high haptoglobinemia [2, 82]. Comparable findings in other species show that infections and metabolic diseases drive inflammation and stimulate de novo SL synthesis [43, 83–85]. Cer, like LPS, binds to Toll-like receptor 4 (TLR4), leading to cytokine production and worsening inflammation [42]. The major impact of Cer, SM, and GlyCer in the pro-inflammatory and pro-resolution phases of LPS/TLR4 activation in macrophages has been reviewed in [44]. Very little data is available in farm animals, but results in humans and rodents reveal that pro-inflammatory cytokines, such as TNF-α and IL-1ß, activate neutral sphingomyelinase, creating a feed-forward loop that exacerbates inflammation [44].

Analysis of Cer composition revealed distinct shifts in chain-length ratios between Hap and Con cows. The enrichment in very long-chain Cer (C22–24, C26) relative to long-chain Cer (C14–C16) indicates differential regulation of CerS, with CerS2 producing very long-chain Cer and CerS5/6 producing shorter-chain Cer [10, 12]. Increased C22–24:C16 and C26:C14 ratios in Hap cows may contribute to inflammatory signaling [18]. Recent studies in IL-10-deficient mice demonstrated that inflammation correlates with elevated very long-chain saturated Cer, and deletion of CerS2 dampens pro-inflammatory gene expression [86]. Moreover, altered long-chain:very long-chain Cer and HexCer ratios have been correlated with the severity of chronic inflammatory diseases in humans [87]. The impact of Cer on specific and non-specific defense mechanisms has been the subject of literature reviews [88, 89]. Although most of the studies available to date have been conducted in humans and rodent models, the structural and functional roles of SL in different species are generally highly conserved [9]. For example, similar roles for Cer have been identified in plants, fungi, and animal cells during stress processes [90].

Changes in Cer ratios may also involve ceramidase activity. Acid ceramidase preferentially hydrolyzes C14–C18 Cer, neutral ceramidase targets C16–C18, while alkaline ceramidases act on unsaturated and very long-chain Cer [11, 91]. The marked reduction of 18:1/14:0 Cer observed here is consistent with enhanced acid ceramidase activity, whereas decreases in 18:1/18:1, 18:1/24:1, and 18:1/25:1 may reflect elevated alkaline ceramidase activity. Interestingly, reduction of 18:1/18:1 Cer was highly significant and aligns with protective effects against oxidative stress in liver ischemia/reperfusion injury [92]. Conversely, LPS downregulates alkaline ceramidase 3, increasing 18:1/18:1 Cer and reducing pro-inflammatory cytokine production [93]. Overall, ceramidases critically regulate inflammation and apoptosis. Increased activity promotes So and So1P formation, protecting against Cer cytotoxicity, whereas reduced activity enhances susceptibility to infection [11, 26, 33, 94, 94–96].

SM levels and the Cer:SM ratio were largely unchanged, except for decreased ratios in C25–C26 species, while LysoSM:SM ratios were elevated. These findings suggest reduced sphingomyelinase activity [97]. Such inhibition contrasts with the common activation of sphingomyelinases during inflammatory processes of genetic, metabolic, or infectious origin [14, 98–100]. Notably, many pathogens specifically target acid sphingomyelinase to alter membrane signaling and facilitate host entry or cytotoxicity [100–102]. The observed inhibition of sphingomyelinases, together with evidence of ceramidase induction, supports the hypothesis that the SL alterations in Hap cows are more likely metabolic responses linked to uterine involution and early lactation, rather than sub-clinical bacterial metritis.

HexCer, and especially LacCer, were increased in Hap cows. HexCer are formed via glucosyl transferase activity on Cer, while LacCer result from lactosyl transferase activity on HexCer (Fig. 1). In other species, HexCer effects on inflammation vary by tissue and pathology [103, 104]. In contrast, LacCer are widely recognized as pro-inflammatory and pro-oxidant mediators in metabolic and infectious disorders [17, 83]. Their action involves phospholipase A2 activation, releasing polyunsaturated fatty acids, which undergo oxidation to generate oxylipins—potent mediators of inflammation [19]. This mechanism may also underlie the decrease in the insat:sat ratio observed in Cer and SM [40, 105]. Although LacCer play roles in pathogen recognition and immune signaling, most reported LacCer changes occur in chronic non-infectious inflammation [17, 106–108].

### Impact of ketosis on the sphingolipidome

The impact of ketosis on the plasma sphingolipidome was modest. The specific role of d18:1P will be discussed separately. Previous work under hyperketogenic diets demonstrated that ketosis elevates Cer in muscle, and to a lesser extent in plasma, driving insulin resistance via mechanisms similar to diabetes [40, 109]. Comparable Cer accumulation has also been observed in adipose tissue [110]. The absence of elevated plasma Cer in Ket cows here may reflect the timing of sampling—on average 7 d postpartum—whereas most studies linking Cer with insulin resistance were conducted prepartum [34].

In Ket cows, DHSM and DHSM:SM ratios were the main variables increased, consistent with prior reports in subclinical ketosis [110]. The rise in 18:0/18:0 and SM18:0/16:0 ratios partially agrees with findings in prepartum ketogenic diet models [109]. Collectively, these results point to elevated de novo SL synthesis (Fig. 1), a process implicated in non-alcoholic steatohepatitis in humans [42], reinforce the hypothesis that disrupted SL synthesis contributes to ketosis pathophysiology in cows [34].

Several LacCer species were also identified as VIP and elevated in Ket cows, potentially fostering chronic inflammation [40]. Muscle and hepatic inflammation could modify the plasma sphingolipidome, which has been proposed in humans as a biomarker of metabolic syndrome [111]. However, inflammatory activity was insufficient to elevate haptoglobinemia, consistent with recent evidence showing oxidative stress in adipose tissue during subclinical ketosis without increased haptoglobinemia [110].

Finally, although not statistically significant, decreases in plasma SM C23–26 were observed only in unsaturated species, proportional to the degree of unsaturation (Fig. 5). This pattern is consistent with reported reductions in SM18:1/24:3 in subketotic cows [110] and may reflect similar oxidative and inflammatory mechanisms as described in Hap cows.

### Impact of mastitis on the sphingolipidome

For this study, cows exhibiting no clinical signs such as udder inflammation or milk clots or elevated haptoglobin levels were selected for SL analysis in the mastitis group. In France, the etiological agents of this type of mastitis are primarily *Staphylococcus aureus* and *Streptococcus uberis* [112]. Mastitis altered the plasma sphingolipidome primarily through an increase in m18:1 and a decrease in LysoSM. DHSM and C25–C26 SM showed a tendency to increase, while Cer and GlyCer were minimally affected. Interestingly, although not significant, unsaturated SM were not decreased in this group and even showed a tendency to increase. Previous analyses of SL in milk during mastitis revealed a global decrease, with LacCer as the notable exception, which was increased [110]. Though results in plasma and milk seem contradictory, they align with prior human studies that revealed contrasting variations in SL in tissues and plasma. For example, in endometriosis, Cer, LacCer, and SM were decreased in tissue but increased in plasma. Only HexCer showed variations in concentration in the same direction, but the effect varies according to analyte size [113]. Similarly, although SL analysis is increasingly used for diagnosis and prognosis in human neurodegenerative diseases, variations in SL concentrations in the brain do not correspond to the same variations in plasma [114]. Additionally, while variations in SL concentration are observed in peritoneal fluid and plasma in mice after the induction of non-septic inflammatory peritonitis via LPS administration, analyte concentration differs in these two matrices over time [83]. Collectively these results suggest the modest quantitative alterations observed in plasma SL in the Mas group likely reflect the predominantly localized nature of the inflammatory process within the udder, with only limited spillover of SL into the circulation. Future studies in milk are therefore necessary to assess the effect of mastitis on SL. These future studies should include an analysis of the different causes of mastitis. Because the severity of the infection affects the host's inflammatory response and the binding of different pathogen motifs to TLRs varies, variations in the SL profile depending on etiology are expected [115].

### Correlations between cytokines and sphingolipids

Significant positive correlations emerged between cytokines and d18:1P in the Hap and Ket groups. This is noteworthy, as d18:1P plays central roles in both infectious and metabolic inflammation [25, 26]. In the Hap group, d18:1P correlated positively with IL-1a and TNFα, consistent with its role in promoting M1 macrophage activation via S1PR3, enhancing inflammatory responses and TNFα secretion. At the same time, d18:1P correlated with IL-10, reflecting its ability to drive macrophage polarization toward the anti-inflammatory phenotype (known as M2 or M2-like) through S1PR1 signaling [28, 29]. In the Ket group, d18:1P correlated strongly with CXCL8 and CCL2, with CCL2 showing the greatest number of SL correlations overall. This aligns with findings in murine macrophages where S1PR4 signaling enhanced CCL2 production [30].

Positive correlations between cytokines and DHCer, Cer, and DHSM were observed in the Con, Hap, and Ket groups. Cer are established mediators of inflammation, driving NF-κB activation, NLRP3 inflammasome assembly, and cytokine release [116]. Elevated DHCer and DHSM, though less studied, indicate activation of de novo SL synthesis and subsequent Cer production (Fig. 1). Their production can be induced by LPS, zymosan and inflammation of metabolic origin [42, 43, 83]. In the Ket group, LacCer also correlated with cytokines, consistent with its role in signaling cascades that amplify macrophage activation and cytokine production [17, 83, 103]. Notably, these correlations primarily involved proinflammatory cytokines and chemokines, which is of particular interest given that elevated β-hydroxybutyrate impairs neutrophil chemotaxis and predisposes to immune dysfunction [117]. This reinforces the proposed use of CXCL8 as an additional marker for ketosis [74].

Fatty acyl chain length appeared to critically influence correlations. Numerous proinflammatory cytokines and chemokines correlated with C18–C20 Cer, whereas C22–C24 Cer showed no association, and C16 Cer displayed weak correlations restricted to the Ket group. C18–C20 Cer are mainly synthesized by CerS4, while C22–C24 Cer derive from CerS2 and C16 Cer from CerS5/6 [12]. CerS2, 5, and 6 are abundantly expressed in immune-related tissues, whereas CerS4 is implicated in T-cell regulation [118] and may be upregulated in adipose tissue and macrophages during metabolic overload [119, 120]. Thus, the strong correlations with C18–C20 Cer may highlight a central role for CerS4 in regulating postpartum inflammation in cows.

Analysis of chemokine–Cer correlations revealed stage-specific inflammatory dynamics. In Hap cows, CCL3 (MIP-1α) and CCL4 (MIP-1β) were the main correlated chemokines. Both are produced by macrophages and activated T cells, drive monocyte/macrophage/NK recruitment, and are typically elevated in chronic inflammation [6, 121]. Together with the strong d18:1P–IL-10 correlations, this suggests that inflammation in Hap cows was progressing toward resolution, consistent with the hypothesis that hyperhaptoglobinemia reflected delayed involution rather than overt metritis. In Ket cows, CXCL8 and CCL2 correlated strongly with Cer. CXCL8 promotes neutrophil recruitment, while CCL2 recruits monocytes and dendritic cells, indicating acute-phase inflammation characterized by early neutrophil and monocyte influx [6, 122]. Coupled with strong correlations involving DHSL and LacCer, these patterns suggest early inflammatory activation in Ket cows, which aligns with the average sampling time (7 d postpartum) and the typically prolonged duration of ketosis [123].

By contrast, cytokine–SM correlations were fewer and predominantly negative, consistent with the role of SM hydrolysis in generating proinflammatory Cer (Fig. 1) [99, 102]. Intact SM, by contrast, stabilize membranes and regulate PLA2 activity [17]. The absence of elevated LysoSM:SM or Cer:SM ratios suggests minimal SM hydrolysis in this study. Interestingly, in the Ket group, positive correlations emerged between several SM such as SM18:1/22:2, SM18:1/24:3, SM18:1/24:2, SM18:1/24:1, SM18:1/25:0, SM18:1/26:2, and SM18:1/26:3 and cytokines including IL-1α, IL-6, IL-2, CXCL8, and CCL2. Of the 10 positive cytokine–SM correlations, 9 involved unsaturated or polyunsaturated SM. Since unsaturated SM were decreased in Ket cows, these findings suggest that their cytokine associations may reflect distinct mechanisms from the negative correlations observed in Hap cows. Broader lipidomic effects, potentially involving fatty acid composition, could contribute to ketosis pathophysiology. Indeed, saturated fatty acids are potent inducers of de novo SL synthesis and NASH [85], whereas unsaturated fatty acids can drive macrophage death, exacerbating obesity-related inflammation [124].

In contrast, very few correlations were detected in the Mas group, which can be attributed to the deliberate exclusion of cows with elevated haptoglobinemia to isolate mastitis-specific effects. Consequently, any local changes in cytokine or SL concentrations during udder infection likely remained moderate and insufficient to cause marked systemic diffusion [70–81].

## Conclusions

Analysis of the effects of acute inflammation on the plasma sphingolipidome in lactating cows seven days after calving demonstrates substantial alterations. The most notable changes involved DHSL, Cer, and LacCer, indicative of heightened de novo SL synthesis and activation of both ceramidase and lactosylceramide synthases. These modifications appear more likely linked to non-infectious processes associated with genital tract lesions rather than infectious mechanisms such as metritis. Similar patterns were observed in ketosis, although the SL alterations were less pronounced. Correlations between SL and cytokines further support the predominance of non-infectious mechanisms, suggesting that hyperhaptoglobinemia was entering a resolution phase, whereas ketosis reflected an earlier stage of inflammatory evolution. Notably, strong correlations between cytokines and chemokines with C18 and C20 Cer suggest a key regulatory role for CerS4 in controlling the inflammatory response. In contrast, the impact of mastitis on the plasma sphingolipidome was quantitatively minor, albeit detectable qualitatively, with no major correlations between cytokines and SL. The absence of systemic SL effects in mastitis is likely due to the selection of cows without overt clinical disease, aside from elevated milk cell counts associated with chronic mastitis. Further studies are warranted to delineate the temporal dynamics of these alterations and to elucidate the underlying mechanisms driving these sphingolipid changes.

## Supplementary Information

Additional file 1: Table S1: Values of variables used to form experimental groups; Table S2: MRM parameters and retention times of sphingolipids quantified in this study; Table S3: Linearity of the method measured for the sphingolipids available as standards; Table S4: Intra-day recovery data of the internal standards of sphingolipids measured in the plasma; Table S5: Effect of age, rank of lactation, body condition score and pathologies on sphingolipids and cytokines; Table S6: Significative interactions between pathologies, age, rank of lactation and body condition score on sphingolipids; Table S7: Descriptive statistics of haptoglobin, ketosis and mastitis on the cytokins; Table S8: Descriptive statistics of haptoglobin, ketosis and mastitis on the sphingolipids; Table S9: Effect of haptoglobin, ketosis and mastitis on the sphingolipids ratio; Table S10: Sphingolipid-cytokine Pearson correlation matrixes and their *P* value.

Additional file 2: Fig. S1: Explanation of how to use the Grubbs test; Fig. S2: Statistical analysis strategies for raw data and ratios; Fig. S3: Sphingolipids dosed seven days after parturition in the plasma of control and pathological cows with and without extreme values; Fig. S4: Sphingolipids dosed seven days after parturition in the plasma of control and hyperhaptobinemia cows; Fig. S5: Sphingolipids dosed seven days after parturition in the plasma of control and hyperketonemia cows; Fig. S6: Sphingolipids dosed seven days after parturition in the plasma of control and mastitis cows with and without outliers cows.

## Data Availability

The datasets generated and/or analysed during the current study are available from the corresponding author on reasonable request.

## Abbreviations

BCS: Body condition score
Cer: Ceramide
CerS: Ceramide synthase
Con: Control
DHCer: Dihydroceramid
DHSL: Dihydrosphingolipid
DHSM: Dihydrosphingomyelin
GlyCer: Glycosylceralide
Hap: Haptoglobin
HexCer: MonoHexosylceramide
HPLC–MS/MS: High Performance Liquid Chromatography coupled with Mass Spectrometry
Ket: Ketosis
LacCer: Lactosylceramid
LacSo: Lactosylsphingosine
LysoSM: Lysosphingomyelin
Mas: Mastitis
PLS-DA: Partial least squares discrimination analysis
S1PR: Sphingosin 1P receptor
SB: Sphingoid base
SCC: Somatic cell count
SL: Sphingolipids
SM: Sphingomyelin
So1P or S1P: Sphingosine-1-phosphate
