# Supplementary material for: Targeted analysis of sphingolipids and cytokines in plasma of dairy cows after calving reveals distinct impacts of systemic inflammation, ketosis, and mastitis
Source: J Anim Sci Biotechnol. 2026 Jan 12;17:6. doi: 10.1186/s40104-025-01325-3 (PMC12794485; doi:10.1186/s40104-025-01325-3)
Supplement: Supplementary file 2 — Additional file 2: Fig. S1: Explanation of how to use the Grubbs test; Fig. S2: Statistical analysis strategies for raw data and ratios; Fig. S3: Sphingolipids dosed seven days after parturition in the plasma of control and pathological cows with and without extreme values; Fig. S4: Sphingolipids dosed seven days after parturition in the plasma of control and hyperhaptobinemia cows; Fig. S5: Sphingolipids dosed seven days after parturition in the plasma of control and hyperketonemia cows; Fig. S6: Sphingolipids dosed seven days after parturition in the plasma of control and mastitis cows with and without outliers cows. [file 40104_2025_1325_MOESM2_ESM.docx]

**Figure S1:** The XLSTAT Grubbs’ test was used to exclude the extreme values. Below is an example of the z-score obtained in the Hap group for IL6 (A) and 18:1/14:0 (B). The test excluded the variables numbered "15" and "3" from the IL6 and 18:1/14:0 analyses, respectively.

The Grubbs test used in this study was the two-tailed test that measures the largest absolute deviation from the mean. The test calculates G using the following equation:

G=(Xi−X)s

where “Xi” is the suspected outlier, ‘X’ is the sample mean, and “s” is the standard deviation.

The value “G” is then compared to a critical value based on the sample size considering the significance level “α = 0.05” and the Student's distribution of the variables.

This test was performed only once per variable, which allowed for the exclusion of one or two extreme values at most.


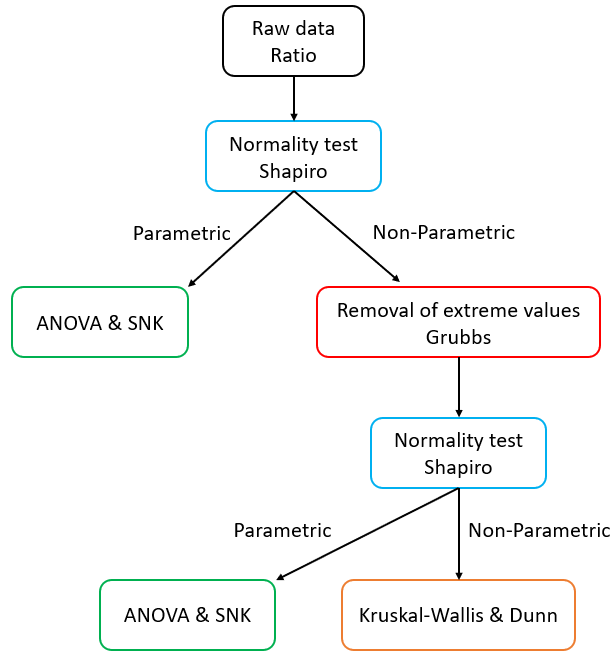


**Figure S2**: Statistical analysis strategies for raw data and ratios, using XLSTAT Biomed software (Addinsoft, Bordeaux, France) and R 4.3.0 ([www.r-project.org](http://www.r-project.org))

**Figure S3**: Partial least squares discriminant analysis (PLS-DA) conducted on sphingolipids dosed seven days after parturition in the plasma of control cows (Con), cows with haptoglobinemia exceeding 400 µg/mL (Hap), cows with a ketosis score greater than or equal to 3 (Ket), cows with milk cell count above 300,000 cells/mL (Mas). (A, B) PLS-DA obtained on all the values. (C, D) PLS-DA obtained without extreme values (Grubbs). (A, C) Discrimination between the four groups of cows on the factor axes extracted from the original explanatory variables. (B, D) Model quality and confusion matrix.

**Figure S4**: Sphingolipids dosed seven days after parturition in the plasma of control cows (Con) and cows with haptoglobinemia exceeding 400 µg/mL (Hap). (A) Partial least squares discriminant analysis showing discrimination between the two groups of cows on the factor axes extracted from the original explanatory variables. (B) Model quality and confusion matrix. (C) Variable Important in the Projection (VIP) on the first component. (D) Variable Important in the Projection (VIP) on the second component.

**Figure S5**: Sphingolipids dosed seven days after parturition in the plasma of control cows (Con) and cows with a ketosis score greater than or equal to 3 (Ket). (A) Partial least squares discriminant analysis showing discrimination between the two groups of cows on the factor axes extracted from the original explanatory variables. (B) Model quality and confusion matrix. (C) Variable Important in the Projection (VIP) on the first component. (D) Variable Important in the Projection (VIP) on the second component.

**Figure S6**: Partial least squares discriminant analysis (PLS-DA) conducted on sphingolipids dosed seven days after parturition in the plasma of control cows (Con) and cows with milk cell count above 300,000 cells/mL (Mas). PLS-DA obtained on (A, B) all the cows and (C-E) after removing 4 cows. (A, C) Discrimination between the two groups of cows on the factor axes extracted from the original explanatory variables and with all the cows. (B) A priori, a posteriori classification, and ranking scores for the two groups of cows. (D) Model quality and confusion matrix. (E) Variable Important in the Projection (VIP) on the first component. (F) Variable Important in the Projection (VIP) on the second component.
